# Identifying Dementia Care Partner Supports as a Public Health Priority: Addressing Equity and Unmet Population Health Needs

**DOI:** 10.1002/alz.089580

**Published:** 2025-01-09

**Authors:** Jenn Hollandsworth Reed, Sherril B Gelmon, Walter D Dawson

**Affiliations:** ^1^ OHSU‐PSU School of Public Health, Portland, OR USA; ^2^ NIA‐Layton Aging & Alzheimer’s Disease Center, Portland, OR USA

## Abstract

**Background:**

The wellbeing of over 11 million care partners of people living with Alzheimer’s disease and related dementias (ADRD) in the United States is increasingly recognized as a public health priority. Addressing care partners’ needs for support and services is particularly important, as care partner burden is associated with negative health outcomes in care partners and people living with ADRD. Care partners from marginalized populations experience inequities in research participation, care burden, and access to supports and services. The goals of this study were 1) to determine supports needed by care partners of people living with ADRD, by empowering population groups historically and currently underserved in ADRD research and services; and 2) to recommend public health policies and interventions to improve care partner wellbeing through social supports and services.

**Methods:**

Caregiving‐related priorities of care partners from four populations underserved in ADRD research and services (Asian, Black, Indigenous, and Latinx Americans) were determined using 5 focus groups and 24 in‐depth interviews with care partners and organizations that support people living with dementia in Oregon. Key governmental and nonprofit organizational leaders (N = 40) participated in a modified Delphi process to identify priorities for supporting care partners through policies and programs. Data were collected from October 2022 through August 2023.

**Results:**

Care partners were generally satisfied with clinical ADRD care but identified opportunities for new supports and improved equity in access to services. Eight themes relating to care partner needs were identified: supports, programs and services, information resources, care coordination, technology, financing, workforce development, and policy. Specific needs varied across populations due to cultural traditions, systemic inequities, and structural racism.

**Conclusions:**

Supporting care partners is a public health strategy that can improve personal wellbeing of care partners and reduce or delay institutional living for people living with ADRD. To ensure equitable supports across diverse populations, policymakers at all levels of government must engage with care partners from marginalized communities to enhance and expand access to services, especially culturally appropriate services. Policies and programs should also be leveraged to address the social determinants of health for care partners in an equitable way.

